# Supplementary material for: Iron Transporter Protein Expressions in Children with Celiac Disease
Source: Nutrients. 2021 Feb 27;13(3):776. doi: 10.3390/nu13030776 (PMC7997288; doi:10.3390/nu13030776)
Supplement: Supplementary file 1 [file nutrients-13-00776-s001.pdf]

nutrients

Supplementary Table S1. Specific characteristics of the staining procedures

Target	Antibody	Host	Dilution	Incubation	Secondary antibody
Duodenal cytochrome B	Anti-CYBRD1, HPA014757, Atlas Antibodies, Stockholm, Sweden	Rabbit	1:800	Overnight	Bionylated anti-rabbit
Divalent metal transporter 1	Anti-DMT1, ab55812, Abcam, Cambridge, UK	Rabbit	1:150	Overnight	ImmPRESS peroxidase Anti rabbit lg
Ferroportin	SLC40A1 Antibody, PA5-22993, Thermo Fisher Scientific, Rockford, USA	Rabbit	1:700	Overnight	ImmPRESS peroxidase Anti rabbit lg
Hephaestin	Hephaestin (L-20), sc-49969, Santa Cruz Biotechnology, Dallas, USA	Goat	1:400	Overnight	Bionylated anti-goat antibody
Transferrin receptor 1	Transferrin Receptor / CD71 Antibody (H68.4), Invitrogen, Thermo Fisher, Rockford, USA	Mouse	1:150	Overnight	ImmPRESS peroxidase Anti rabbit lg

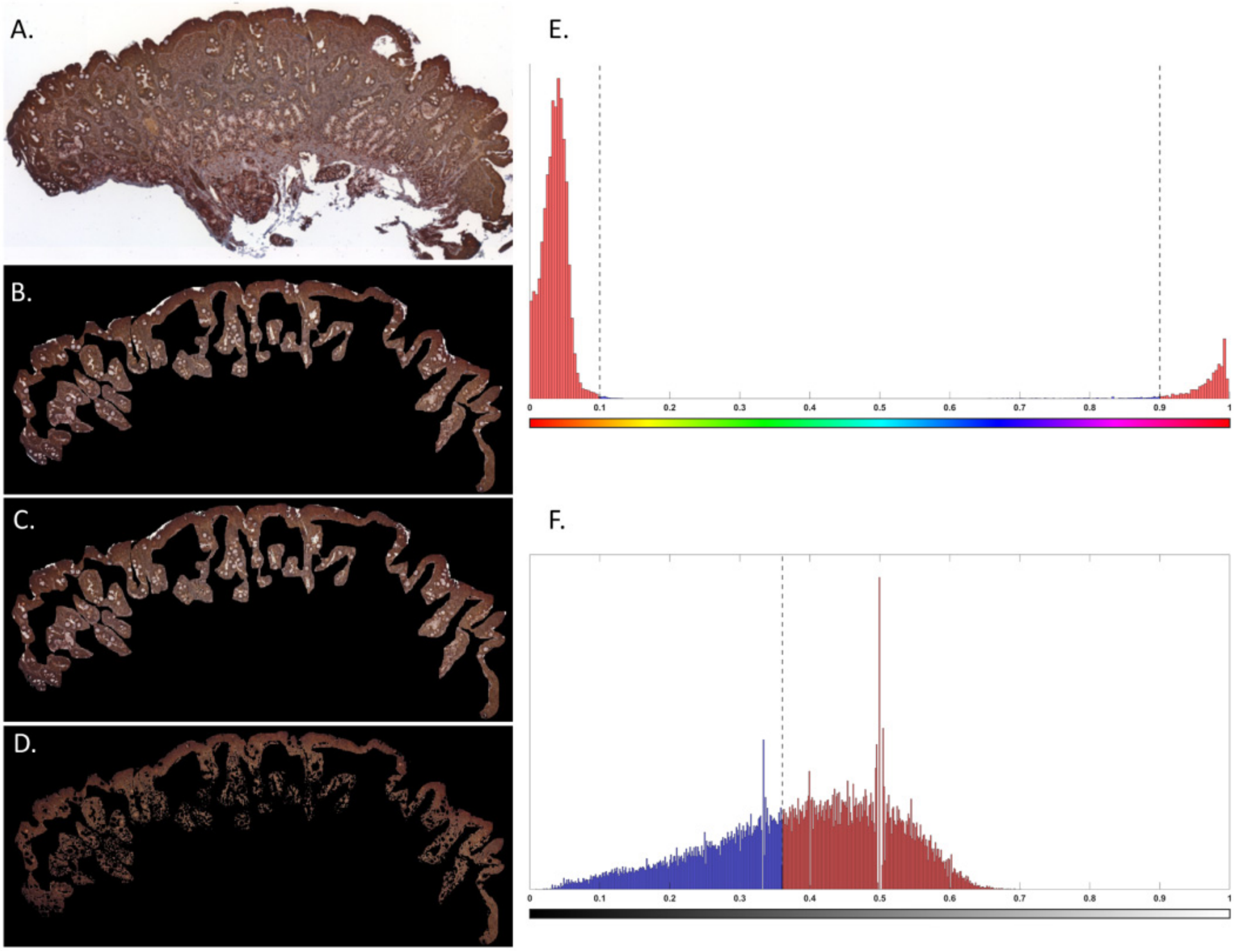

Figure S1. The process for measuring the saturation and stained area of iron transporter proteins in enterocytes as exemplified by ferroportin staining in a patient with subtotal villous atrophy and anemia. (**A**) The scanned ferroportin stained section. (**B**) The epithelial layer free hand selected using Fiji Image J program. (**C**) The red color chosen from the hue channel. (**D**) The saturation thresholded to show the area of the ferroportin staining. (**E**) A histogram of the hue channel, where the red color is chosen. (**F**) A histogram of the saturation channel.

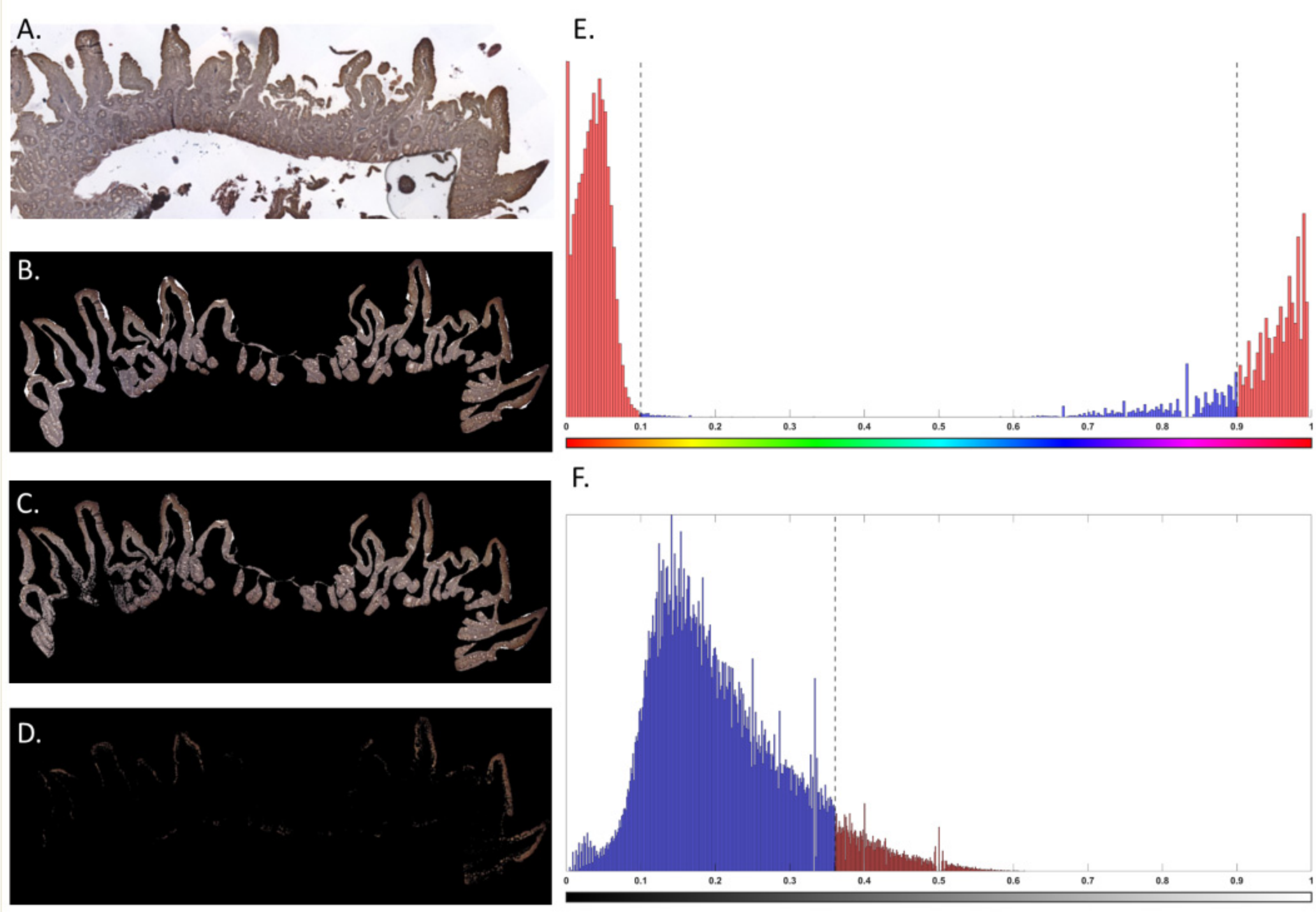

Figure S2. The process for measuring the saturation and stained area of iron transporter proteins in enterocytes as exemplified by ferroportin staining in a control child with anemia. (**A)** The scanned ferroportin stained section. (**B**) The epithelial layer free hand selected using Fiji Image J program. (**C**) The red color chosen from the hue channel. (**D**) The saturation thresholded to show the area of the ferroportin staining. (**E**) A histogram of the hue channel, where the red color is chosen. (**F**) A histogram of the saturation channel.
